# Neonatal mortality within 24 hours of birth in six low- and lower-middle-income countries

**DOI:** 10.2471/BLT.15.160945

**Published:** 2016-08-30

**Authors:** Abdullah H Baqui, Dipak K Mitra, Nazma Begum, Lisa Hurt, Seyi Soremekun, Karen Edmond, Betty Kirkwood, Nita Bhandari, Sunita Taneja, Sarmila Mazumder, Muhammad Imran Nisar, Fyezah Jehan, Muhammad Ilyas, Murtaza Ali, Imran Ahmed, Shabina Ariff, Sajid B Soofi, Sunil Sazawal, Usha Dhingra, Arup Dutta, Said M Ali, Shaali M Ame, Katherine Semrau, Fern M Hamomba, Caroline Grogan, Davidson H Hamer, Rajiv Bahl, Sachiyo Yoshida, Alexander Manu

**Affiliations:** a International Center for Maternal and Newborn Health, Johns Hopkins University, Baltimore, United States of America (USA).; b Division of Population Medicine, Cardiff University School of Medicine, Cardiff, Wales.; c Department of Population Health, London School of Hygiene and Tropical Medicine, London, England.; d School of Paediatrics and Child Health, University of Western Australia, Perth, Australia.; e Centre for Health Research and Development, Society for Applied Studies, New Delhi, India.; f Department of Paediatric and Child Health, The Aga Khan University, Karachi, Pakistan.; g Center for Public Health Kinetics, New Delhi, India.; h Public Health Laboratory Ivo de Carneri, Pemba, United Republic of Tanzania.; i BetterBirth Program, Ariadne Labs, Boston, USA.; j Center for Global Health and Development, Boston University, Boston, USA.; k Department of Maternal, Newborn, Child and Adolescent Health, World Health Organization, avenue Appia 20, 1211 Geneva 27, Switzerland.

## Abstract

**Objective:**

To estimate neonatal mortality, particularly within 24 hours of birth, in six low- and lower-middle-income countries.

**Methods:**

We analysed epidemiological data on a total of 149 570 live births collected between 2007 and 2013 in six prospective randomized trials and a cohort study from predominantly rural areas of Bangladesh, Ghana, India, Pakistan, the United Republic of Tanzania and Zambia. The neonatal mortality rate and mortality within 24 hours of birth were estimated for all countries and mortality within 6 hours was estimated for four countries with available data. The findings were compared with published model-based estimates of neonatal mortality.

**Findings:**

Overall, the neonatal mortality rate observed at study sites in the six countries was 30.5 per 1000 live births (range: 13.6 in Zambia to 47.4 in Pakistan). Mortality within 24 hours was 14.1 per 1000 live births overall (range: 5.1 in Zambia to 20.1 in India) and 46.3% of all neonatal deaths occurred within 24 hours (range: 36.2% in Pakistan to 65.5% in the United Republic of Tanzania). Mortality in the first 6 hours was 8.3 per 1000 live births, i.e. 31.9% of neonatal mortality.

**Conclusion:**

Neonatal mortality within 24 hours of birth in predominantly rural areas of six low- and lower-middle-income countries was higher than model-based estimates for these countries. A little under half of all neonatal deaths occurred within 24 hours of birth and around one third occurred within 6 hours. Implementation of high-quality, effective obstetric and early newborn care should be a priority in these settings.

## Résumé

**Objectif:**

Estimer la mortalité néonatale, en particulier dans les 24 heures suivant la naissance, dans six pays à revenu faible et intermédiaire - tranche inférieure.

**Méthodes:**

Nous avons analysé des données épidémiologiques portant sur un total de 149 570 naissances vivantes qui avaient été recueillies entre 2007 et 2013 lors de six essais prospectifs randomisés et d'une étude de cohorte réalisés dans des zones majoritairement rurales du Bangladesh, du Ghana, d'Inde, du Pakistan, de Tanzanie et de Zambie. Le taux de mortalité néonatale ainsi que la mortalité dans les 24 heures suivant la naissance ont été estimés pour tous ces pays; la mortalité dans les 6 heures suivant la naissance a été estimée pour quatre pays sur lesquels nous avions des données. Les résultats ont été comparés aux estimations de la mortalité néonatale publiées, qui ont été établies d'après des modèles.

**Résultats:**

Globalement, le taux de mortalité néonatale observé sur les sites étudiés dans les six pays était de 30,5 pour 1000 naissances vivantes (de 13,6 en Zambie à 47,4 au Pakistan). La mortalité globale dans les 24 heures suivant la naissance était de 14,1 pour 1000 naissances vivantes (de 5,1 en Zambie à 20,1 en Inde) et 46,3% de l'ensemble des décès néonataux étaient survenus dans les 24 heures (de 36,2% au Pakistan à 65,5% en Tanzanie). Le taux de mortalité dans les 6 heures suivant la naissance était de 8,3 pour 1000 naissances vivantes, soit 31,9% de la mortalité néonatale totale.

**Conclusion:**

La mortalité néonatale dans les 24 heures suivant la naissance dans des zones majoritairement rurales de six pays à revenu faible et intermédiaire-tranche inférieure était supérieure aux estimations établies d'après des modèles pour ces pays. Un peu moins de la moitié des décès néonataux étaient survenus dans les 24 heures suivant la naissance et environ un tiers dans les 6 heures. La mise en place de soins obstétriques et néonataux efficaces et de haute qualité devrait être une priorité dans ces pays.

## Resumen

**Objetivo:**

Calcular la mortalidad neonatal, especialmente durante las 24 horas posteriores al nacimiento, en países con ingresos bajos y países con ingresos medios más bajos.

**Métodos:**

Se analizaron datos epidemiológicos de un total de 149 570 nacidos vivos recopilados entre 2007 y 2013 en seis ensayos aleatorizados prospectivos y un estudio de cohortes de zonas principalmente rurales de Bangladesh, Ghana, India, Pakistán, la República Unida de Tanzanía y Zambia. Se calculó la tasa de mortalidad neonatal y la mortalidad durante las 24 horas posteriores al nacimiento en todos los países y se estimó la tasa de mortalidad en 6 horas en cuatro países con información disponible. Los resultados se compararon con estimaciones publicadas basadas en modelos de mortalidad neonatal.

**Resultados:**

En general, la tasa de mortalidad neonatal observada en los lugares de estudio de los seis países fue de 30,5 por cada 1 000 nacidos vivos (alcance: 13,6 en Zambia a 47,4 en Pakistán). En conjunto, la mortalidad durante las primeras 24 horas fue de 14,1 por cada 1 000 nacidos vivos (alcance: 5,1 en Zambia a 20,1 en India) y el 46,3% de todas las muertes neonatales se produjo durante las primeras 24 horas (alcance: 36,2% en Pakistán a 65,5% en la República Unida de Tanzanía). La mortalidad en las primeras 6 horas fue de 8,3 por cada 1 000 nacidos vivos, es decir, un 31,9% de mortalidad neonatal.

**Conclusión:**

La mortalidad neonatal durante las 24 horas posteriores al nacimiento en zonas principalmente rurales de seis países con ingresos bajos y países con ingresos medios más bajos fue superior a las estimaciones basadas en modelos realizadas para estos países. Algo menos de la mitad de todas las muertes neonatales se produjeron durante las 24 horas posteriores al nacimiento y cerca de un tercio durante las primeras 6 horas de vida. En estos lugares, la implementación de obstetricia eficaz y de alta calidad y la atención a recién nacidos debería ser una prioridad.

## ملخص

**الغرض:**

تقدير معدل وفيات الأطفال حديثي الولادة، وبالأخص في غضون 24 **  **ساعة من الولادة، في ستة بلدان تندرج ضمن فئات الدخل المنخفض والشريحة الدنيا من فئة الدخل المتوسط.

**الطريقة:**

قمنا بتحليل البيانات الوبائية لإجمالي 149,570 **  **من المواليد على قيد الحياة وتم جمعها في الفترة ما بين عام 2007 وعام 2013 في ست تجارب عشوائية استباقية ودراسة أترابية من المناطق الريفية الرئيسية في بنغلاديش وغانا والهند وباكستان وجمهورية تنزانيا المتحدة وزامبيا. تم تقدير معدل وفيات الأطفال حديثي الولادة والوفيات في غضون 24 ساعة من الولادة لجميع البلدان وتم تقدير معدل الوفيات في غضون 6 ساعات لأربعة بلدان تتوفر لها البيانات. وتمت مقارنة النتائج بالتقديرات المنشورة المستندة إلى النموذج لمعدل وفيات الأطفال حديثي الولادة.

**النتائج:**

عمومًا، كان معدل وفيات الأطفال حديثي الولادة الذي لوحظ في مواقع الدراسة في ستة بلدان يبلغ 30.5 لكل 1000 من المواليد على قيد الحياة (نطاق القياس: 13.6 في زامبيا إلى 47.4 **  **في باكستان). وكان معدل الوفيات في غضون 24 **  **ساعة يبلغ 14.1 لكل 1000 من المواليد على قيد الحياة عمومًا (نطاق القياس: 5.1 في زامبيا إلى 20.1 **  **في الهند) وحدثت جميع حالات الوفاة للأطفال حديثي الولادة بنسبة 46.3‏% في غضون 24 **  **ساعة (نطاق القياس: بنسبة 36.2‏% في باكستان إلى 65.5‏% في جمهورية تنزانيا المتحدة). بلغ معدل الوفيات في أول 6 **  **ساعات 8.3 لكل 1000 من المواليد على قيد الحياة، أي بنسبة 31.9‏% من وفيات الأطفال حديثي الولادة.

**الاستنتاج:**

إن معدل الوفيات للأطفال حديثي الولادة في غضون 24 **  **ساعة من الولادة في المناطق الريفية الرئيسية في ستة بلدان تندرج ضمن فئات الدخل المنخفض والشريحة الدنيا من فئة الدخل المتوسط كان أعلى من التقديرات المستندة إلى النموذج الخاصة بهذه البلدان. حدثت الوفيات للأطفال حديثي الولادة بمعدل أقل من النصف قليلاً في غضون 24 **  **ساعة من الولادة، وحدثت ثلث الوفيات تقريبًا في غضون 6 **  **ساعات من إجمالي جميع حالات الوفاة للأطفال حديثي الولادة. ينبغي أن يمثل تنفيذ الإجراءات الفعالة وعالية الجودة للرعاية التوليدية والرعاية المبكرة للأطفال حديثي الولادة أولوية في هذه الأماكن. ****

## 摘要

**目的:**

旨在评估 6 个中低收入国家的新生儿死亡率、尤其是出生后 24 小时内的死亡率。

**方法:**

我们一共对 149 570 名新出生婴儿的流行病学数据进行了分析，该数据来自于 2007 年至 2013 年对巴基斯坦、印度、加纳、孟加拉国、坦桑尼亚联合共和国及赞比亚主要乡村地区进行的 6 项前瞻性随机试验和一项队列研究。 我们对所有国家的新生儿死亡率以及新生儿出生后 24 小时内的死亡率进行了评估，并对拥有可用数据的 4 个国家的新生儿出生后 6 小时内的死亡率进行了评估。 我们将调查结果与已发布的、基于模型的新生儿死亡率估算结果进行了比较。

**结果:**

总体而言， 6 个国家的研究地的实际新生儿死亡率为每 1000 名新出生婴儿中有 30.5 名死亡（范围： 从赞比亚的 13.6 到巴基斯坦的 47.4）。 新生儿出生后 24 小时内死亡率为每 1000 名新出生婴儿中共有 14.1 名死亡（范围： 从赞比亚的 5.1 到印度的 20.1），且 46.3% 的新生儿死亡案例发生在婴儿出生后 24 小时内（范围： 从巴基斯坦的 36.2% 到坦桑尼亚联合共和国的 65.5%）。 婴儿出生后 6 小时内的死亡率为每 1000 名新出生婴儿中有 8.3 名死亡，即新生儿死亡率为 31.9%。

**结论:**

6 个中低收入国家主要乡村地区的新生儿出生后 24 小时内的死亡率均高于这些国家的基于模型的估算结果。 略低于一半的新生儿死亡案例发生在婴儿出生后 24 小时内，而大约三分之一的案例发生在婴儿出生后 6 小时内。 上述国家应将实施优质、有效的产科护理和早期新生儿护理视为工作重点。

## Резюме

**Цель:**

Дать оценку смертности новорожденных, особенно в первые 24 часа жизни, в шести странах с низким уровнем дохода и уровнем дохода ниже среднего.

**Методы:**

Проведен анализ эпидемиологических данных, полученных в результате рождения 149 570 живых детей в промежутке с 2007 по 2013 год, на основании шести проспективных рандомизированных исследований и одного когортного исследования в преимущественно сельских регионах Бангладеша, Ганы, Замбии, Индии, Объединенной Республики Танзании и Пакистана. Были рассчитаны показатели смертности новорожденных в первые 24 часа для всех стран, а оценка смертности в первые шесть часов была получена для четырех стран с доступными данными. Результаты сравнили с опубликованными показателями смертности новорожденных, рассчитанными на основе моделирования.

**Результаты:**

В целом показатель смертности новорожденных, наблюдаемый в центрах проведения исследования шести стран, составил 30,5 на 1000 рождений живых детей (от 13,6 в Замбии до 47,4 в Пакистане). Смертность в течение первых суток в целом составляла 14,1 на 1000 рождений живых детей (диапазон: от 5,1 в Замбии до 20,1 в Индии) или 46,3% от всех смертей новорожденных (от 36,2% в Пакистане до 65,5% в Объединенной Республике Танзания). Смертность в первые шесть часов составляла 8,3 на 1000 рождений живых детей или 31,9% от всех смертей новорожденных.

**Вывод:**

Смертность новорожденных в первые 24 часа в преимущественно сельских регионах шести стран с низким уровнем дохода и уровнем дохода ниже среднего была выше, чем показатели, рассчитанные на основе моделирования для этих же стран. Чуть менее половины всех смертей новорожденных произошли в течение 24 часов после рождения, и около трети произошли в течение первых шести часов. Поэтому внедрение высококачественной эффективной акушерской помощи и ухода за новорожденными должно стать первостепенной задачей.

## Introduction

Neonatal mortality remains unacceptably high and the risk is greatest on the first day of life – these were the conclusions of the 14th annual State of the World’s Mothers report, entitled *Surviving the first day*,^1^ which presented estimates of the risk of maternal, neonatal and first-day deaths in 186 countries for 2011. Worldwide, it was estimated that more than 1 million babies died within 24 hours of birth in 2011, which represented more than one third of all neonatal deaths and 15% of all deaths in children younger than 5 years. The risk was highest in sub-Saharan Africa but the number of deaths was highest in South Asia.

Estimated risks for 2013, which have recently been published, were similar.^2^ The estimates for 2011 and 2013 were both derived using a modelling approach based on data on live births and neonatal deaths from the United Nations Inter-agency Group for Child Mortality Estimation. Although data from vital registration systems were available for 109 countries, only those from 57 countries were judged to be of high quality – the proportion of deaths in the first day of life was calculated directly for these countries. For the other 129 countries without high-quality vital registration system data, the proportion of deaths in the first day of life was estimated from country-specific United Nations’ data using a neonatal survival curve derived from Demographic and Health Survey data for 79 countries (median reporting date: 1999). Model-based estimates provide essential information on countries lacking high-quality direct assessments: they illustrate the magnitude of the problem, enable cross-country comparisons to be made and help guide health policy and health service planning. However, the estimates must be accurate and up-to-date.

For many countries, epidemiological studies have collected prospective, population-based information on pregnancies, births and newborn deaths that could be used to replace model estimates with high-quality, up-to-date data. We obtained such information from community surveillance systems established as part of large, ongoing or recently completed studies in six low- and lower-middle-income countries in sub-Saharan Africa and South Asia: Bangladesh, Ghana, India, Pakistan, the United Republic of Tanzania and Zambia. The aims of this analysis were to estimate the neonatal mortality rate and the percentage of neonatal deaths occurring within the first 6 and 24 hours of life in these countries and to compare the results with model-based estimates.

## Methods

Our analysis involved epidemiological data from population-based, cohort or intervention studies carried out at seven sites in the six countries between 2007 and 2013 and coordinated by the World Health Organization (WHO; Table 1; available at: http://www.who.int/bulletin/volumes/94/10/15-160945) 

**Table 1:** Studies included, analysis of neonatal mortality in six low- and lower-middle-income countries, 2007–2013

Characteristic	Study country						
	Bangladesh	Ghana^a^	India	Pakistan (Karachi)	Pakistan (Sindh)	United Republic of Tanzania	Zambia
Study site	Projahnmo area, Sylhet district	7 rural and semi-urban districts in the Brong-Ahafo Region	18 rural and semi-urban areas in Haryana State	4 peri-urban and 1 urban site in Karachi city	Naushahro Feroze, a rural district of Sindh Province	Pemba Island	45 clusters in Southern Province
Approximate population under surveillance, *n*	182 000	600 000	550 000	274 000	243 000	400 000	ND^b^
Proportion of women who had ≥ 1 antenatal care visit, %	60	97	59	75	78	85	96
Proportion of women with a skilled birth attendant present at delivery, %	14	64	50	59	56	51	55
Study period	2007–2009	2008	2008–2010	2011	2010–2012	2009–2013	2011–2013
Type of study	Cluster randomized trial	Two cluster randomized trials	Cluster randomized trial	Cohort study (Health and Demographic Surveillance system data)	Cluster randomized trial	Individually randomized trial	Cluster randomized trial
Intervention	Chlorhexidine applied to the umbilical cord stump	Low-dose vitamin A supplementation for women of reproductive age (ObaapaVitA trial) and home visits by community health workers (Newhints trial)	Implementation of Integrated Management of Neonatal and Childhood Illness programme, which included home visits for early newborn care	No intervention	Intervention package for mothers and babies implemented by traditional birth attendants and lady health workers	Chlorhexidine applied to the umbilical cord stump	Chlorhexidine applied to the umbilical cord stump
Identification of pregnancy	Noted during home visits carried out by community health workers every 2-months	Noted during home visits carried out by trained field workers every 4 weeks	Noted during monthly home visits by trained study workers	Noted during home visits carried out by community health workers every 3 months	Noted during home visits carried out by community health workers every 3 months	Noted during weekly contacts with families made by community health workers	Pregnant women enrolled during antenatal visits and community outreach programmes
Identification of birth	Noted during a home visit carried out at birth	Noted during home visits carried out by trained field workers every 4 weeks	Noted during monthly home visits by trained study workers or during follow-up visits to pregnant women	Reported by the birth attendant and confirmed by calling the family or noted during a surveillance home visit carried out by a study worker	Reported by the birth attendant and confirmed by calling the family, reported by a lady health worker or village volunteer or noted during a surveillance visit by a study worker	Reported to a central information system by a health worker in the local maternity ward, a maternal and child health worker or a traditional birth attendant	Notified by a staff member at a facility or by a family member at a visit or by phone
Identification of neonatal death	Noted during the 6 home visits made during the neonatal period	Noted during the home visit made during the neonatal period or the home visit made after the neonatal period	Noted during the home visit made at the end of the neonatal period on day 29	Noted during the 6 home visits made during the neonatal period	Noted during the 3 home visits made during the neonatal period or on a quarterly home visit	Noted during the 5 home visits made during the neonatal period	Noted during the 5 home visits made during the neonatal period
Groups included in the analysis	Control clusters only	Full trial cohorts	Control clusters only	Full trial cohort	Control clusters only	Full trial cohort	Control clusters only

ND: not determined.

^a^ For Ghana, data came from a combination of two trials: (i) the ObaapaVitA trial, which collected data between January and October 2008; and (ii) the Newhints trial, which collected data during the preparatory phase in November and December 2008 – the full intervention did not start until June 2009.

^b^ The population covered by the trial in Zambia was not determined because women were enrolled at antenatal clinics or during community outreach.

Intervention studies that recruited newborn babies were excluded as some deaths that occurred before enrolment could have been missed. Data were derived from five cluster randomized trials, one individually randomized trial and one cohort study. Three trials evaluated the effect of applying chlorhexidine to the umbilical cord on neonatal mortality, one examined low-dose vitamin A supplementation for women of reproductive age and three involved home visits by community health workers. Together the trials included over 2 million women in predominantly rural communities. All studies included a community-based surveillance system involving home visits by trained field workers who collected data on vital events, including pregnancies, births, deaths, illness and migration, every one to three months. For the analysis, data from both intervention and control arms were pooled if there was no evidence that the intervention affected neonatal mortality. Otherwise, only data from the control arm were included.

For Bangladesh, the analysis used data from the control arm of a cluster randomized trial evaluating the effect of different umbilical cord cleansing regimens on neonatal mortality and infection.^3^^,^^4^ Between 2007 and 2009, community health workers made home visits every two months. The dates and times of births were recorded soon after birth and the dates and times of deaths were recorded during postnatal visits on days 1, 3, 6, 9, 15 and 28. For Ghana, data on all participants in two cluster randomized trials were used because the interventions had no significant effect on neonatal mortality.^5^^,^^6^ The trials were conducted in seven predominantly rural districts in 2008: one examined the effect of low-dose vitamin A supplementation on maternal and infant mortality and one evaluated the effect of a home visit programme on neonatal mortality and newborn care practices. Trained field workers made home visits every four weeks, at which they recorded births and deaths. For each neonatal death, a verbal autopsy was performed by a trained supervisor, who determined whether or not the death occurred within the first 24 hours. For India, data from the control arm of a cluster randomized trial on the effect of an Integrated Management of Neonatal and Childhood Illness programme on neonatal and infant mortality were used.^7^ The trial was conducted in rural and semi-urban areas of Haryana State between 2008 and 2010. Home visits took place monthly and all households with a live birth were visited on day 29 to document the vital status of the infant. As in Ghana, an independent verbal autopsy was performed for any death.

For Pakistan, data came from a Health and Demographic Surveillance system covering approximately 274 000 people in urban and peri-urban Karachi and from the control arm of cluster randomized trial in Sindh. In Karachi, data were collected by community health workers who visited households every three months in 2011.^8^ In Sindh, the trial investigated the effect of interventions involving traditional birth attendants and Lady Health Workers on birth asphyxia, sepsis and the number of low-birth-weight infants and, consequently, on neonatal mortality. For the United Republic of Tanzania, data from all participants in an individually randomized trial examining the effect of applying chlorhexidine to the umbilical cord stump on neonatal mortality were used because the intervention had no significant effect. The trial was conducted on Pemba Island and involved health-care workers in the local maternity ward, maternal and child health workers and traditional birth attendants. All births were registered centrally and households where a birth occurred were visited by a field supervisor; 90% of visits were made within 12 hours of delivery. The date and time of birth were confirmed with the birth attendant. For Zambia, data from the control arm of a cluster randomized trial on the application of chlorhexidine were used. The trial was conducted in the Southern Province between 2011 and 2013.^9^ As over 95% of women attended antenatal clinics during pregnancy, trial enrolment took place at health centres and during community outreach programmes conducted by health workers. Data on births and deaths were collected through a community-based birth notification system and during home visits after delivery.

### Statistical analysis

At all sites, a neonatal death was defined as a death within the first 28 days of life (i.e. day 0 to day 27). For Bangladesh, India, the United Republic of Tanzania and Zambia, we estimated mortality within the first 24 hours directly using the difference between the time and date of birth and the time and date of death. For Ghana, the occurrence of death within the first 24 hours was determined by verbal autopsy and, in Pakistan, deaths that occurred on the day of birth were taken as deaths that occurred within the first 24 hours. Mortality in the first 24 hours of life was expressed as deaths per 1000 live births and as a percentage of all neonatal deaths. In addition, mortality in the first six hours of life was calculated for the four sites where relevant data were available. For comparison, we used modelling estimates of neonatal mortality, mortality within the first 24 hours of birth and the percentage of all neonatal deaths occurring within 24 hours for each country derived by Oza et al.^2^

## Results

**Fig. 1:**
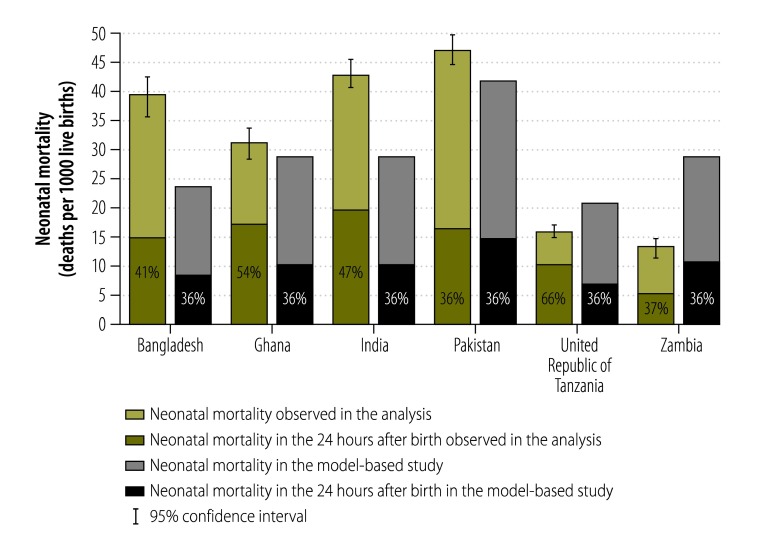
Neonatal mortality, six low- and lower-middle-income countries, 2007–2013

Notes: The percentages in the columns indicate the proportion of all neonatal deaths that occurred in the first 24 hours after birth. Model-based estimates of neonatal mortality and mortality within the first 24 hours of birth were derived by Oza et al.^2^

The total number of live births included in the analysis was 149 570: the number in each country ranged from 11 143 in Bangladesh to 44 450 in the United Republic of Tanzania (Table 2). The pooled neonatal mortality rate across all studies was 30.5 per 1000 live births and country-specific neonatal mortality rates ranged from 13.6 per 1000 live births (95% confidence interval, CI: 12.0–15.3) in Zambia to 47.4 per 1000 (95% CI: 44.9–49.9) in Pakistan. Overall, mortality within the first 24 hours was 14.1 per 1000 live births – the lowest rate was 5.1 per 1000 live births in Zambia and the highest was 20.1 per 1000 live births in India. The percentage of neonatal deaths occurring within the first 24 hours was 46.3% overall: the figure ranged from 36.2% (95% CI: 33.6–38.8) in Pakistan to 65.5% (95% CI: 62.0–69.0) in the United Republic of Tanzania. At the four sites where data on deaths within the first six hours were available, the mortality rate was 8.3 per 1000 live births overall, which corresponded to 31.9% of the overall neonatal mortality rate at these sites. The proportion of neonatal deaths that occurred in the first 6 hours ranged from 17.5% in Zambia to 36.0% in the United Republic of Tanzania (Table 2). Fig. 1 shows a comparison of the neonatal mortality rates we observed in the six study countries with the corresponding rates derived in a modelling-based study by Oza et al.^2^ According to the modelling study, 36% of neonatal deaths would be expected to occur in the first 24 hours of life in our six study countries, which is lower than the average of 46% we observed.

**Table 2:** Live births and neonatal mortality, six low- and lower-middle-income countries, 2007–2013

Parameter	Bangladesh	Ghana	India	Pakistan	United Republic of Tanzania	Zambia	Total
Approximate population under surveillance, *n*	182 000	600 000	550 000	517 000	400 000	ND^a^	> 2 000 000
Study period	2007–2009	2008	2008–2010	2010–2012	2009–2013	2011–2013	NA
Live births during the study period, no.	11 143	15 461	30 920	28 250	44 450	19 346	149 570
Neonatal deaths,^b^ no.	442	484	1 326	1 338	714	263	4 567
Neonatal mortality rate, deaths per 1000 live births (95% CI)	39.7 (36.1–43.5)	31.3 (28.6–34.2)	42.9 (40.7–45.2)	47.4 (44.9–49.9)	16.1 (14.9–17.2)	13.6 (12.0–15.3)	30.5 (29.7–31.4)
Neonatal deaths in the first 24 hours of life, no.	182	263	620	484	468	98	2 115
Neonatal mortality rate in the first 24 hours, deaths per 1000 live births (95% CI)	16.3 (14.1–18.9)	17.0 (15.0–19.2)	20.1 (18.6–21.7)	17.1 (15.7–18.8)	10.5 (9.6–11.5)	5.1 (4.1–6.2)	14.1 (13.5–14.8)
Proportion of neonatal deaths occurring in the first 24 hours, % (95% CI)	41.2 (36.5–45.9)	54.3 (49.8–58.8)	46.8 (44.0–49.5)	36.2 (33.6 38.8)	65.5 (62.0–69.0)	37.3 (31.4–43.4)	46.3 (44.9–47.8)
Neonatal deaths in the first 6 hours of life, no.	113	ND^c^	460	ND^c^	257	46	876
Neonatal mortality rate in the first 6 hours, deaths per 1000 live births (95% CI)	10.1 (8.4–12.2)	ND^c^	14.9 (13.6–16.3)	ND^c^	5.8 (5.1–6.5)	2.4 (1.7–3.2)	8.3 (7.7–8.8)
Proportion of neonatal deaths occurring in the first 6 hours, % (95% CI)	25.6 (21.6–30.0)	ND^c^	34.7 (32.1–37.3)	ND^c^	36.0 (32.5–39.6)	17.5 (13.1–22.6)	31.9 (30.2–33.7)
							

CI: confidence interval; NA: not applicable; ND: not determined.

^a^ The population covered by the trial in Zambia was not determined because women were enrolled at antenatal clinics or during community outreach.

^b^ Neonatal deaths in the first 28 days of life.

^c^ The time of death during the first day was not available for this study.

## Discussion

Our main finding was that, overall, a little under half of all neonatal deaths in the study populations occurred within 24 hours of birth – the figure ranged from 36% to 66% across the study sites – and about one in three neonatal deaths occurred within six hours of birth. Our data confirm that neonatal mortality remains high in many low- and lower-middle-income countries.

Our study has several strengths. The total study population was large and data on almost 150 000 live births and over 4500 neonatal deaths were included. Moreover, as the data came from large, high-quality and prospective surveillance systems that were established to capture population-based data on births and deaths, the numerators and denominators were accurate. In particular, our data were more likely to be complete than data from large country-wide surveys, such as Demographic and Health Surveys, which have, in the past, underestimated the true burden of rare events such as neonatal deaths and stillbirths.^10^ In addition, nationally representative surveys suffer from recall bias, which may be particularly important for deaths occurring very close to birth. Many mothers may report neither the birth nor the death of such babies during surveys. The studies we analysed prospectively identified and followed up all births in the target populations and the possibility that stillbirths were misclassified as very early neonatal deaths was minimized in these studies by active surveillance and careful verbal autopsy.

### Comparison with model-based estimates

Generally the neonatal mortality rates we observed were significantly different from the model-based estimates: the 95% CI for the observed neonatal mortality rate did not include the modelled estimate for any country except Ghana. Moreover, the observed proportion of neonatal deaths occurring in the first 24 hours was significantly higher than the modelled estimate of 36% for all countries except Pakistan and Zambia. In Pakistan, the observed proportion of deaths in the first 24 hours may have been underestimated because, in one trial, only deaths recorded on the date of birth were regarded as having occurred in the first 24 hours. This explanation does not apply to Zambia, however.

Our observation that a higher proportion of neonatal deaths occurred in the first 24 hours than predicted by modelling must be interpreted in the context of the overall neonatal mortality rate. The proportion of neonatal deaths occurring in the first day of life is generally higher when the neonatal mortality rate is low: in 2011, a proportion of 35% was reported in sub-Saharan Africa where the neonatal mortality rate was 34 per 1000 live births compared with a proportion of 67% for developed countries where the neonatal mortality rate was 3 per 1000 live births.^1^ In our study, the observed neonatal mortality rate was higher than the modelled estimate for Bangladesh, Ghana, India and Pakistan but lower for the United Republic of Tanzania and Zambia. Although no special criteria were used to select the study populations, these populations did live in geographical areas with high mortality. Consequently, the neonatal mortality rates in these settings may have been higher than the national averages. Given higher neonatal mortality rates, the proportion of deaths occurring in the first 24 hours would have been expected to be lower, but we found the opposite. This observation has important implications for maternal and newborn health programmes in low- and lower-middle-income countries because most neonatal deaths globally occur in populations similar to our study populations, in which the neonatal mortality rate is likely to be near or above the national average.

The neonatal mortality rates observed in our study would have been expected to be lower than modelled estimates because the data were collected more recently: between 2007 and 2013 compared with a median survey year of 1999 for the data used in the model. However, improvements could have subsequently taken place in the study areas. In particular, there have been many advances in newborn care in recent years in settings where access to adequate health care is not universal. For example, in a large trial in Ghana, home visits by community health workers to promote essential newborn care practices have been shown to reduce neonatal mortality by 12% and have the potential to reduce childhood mortality by 4.9%.^6^ In our study, the observed neonatal mortality rate was lower than the modelled estimate only in the United Republic of Tanzania and Zambia. We believe the higher rates we found in the other four countries were due to differences in methods used: our estimates were based on prospectively defined population groups in which all births were followed up whereas the model’s estimates were based on cross-sectional surveys, which are more prone to recall bias and, consequently, to underestimation of the real neonatal mortality rate. Although the provision of intrapartum and postnatal care by skilled birth attendants is known to be crucial for neonatal survival, we observed no clear relationship between coverage by skilled birth attendants or antenatal care and neonatal mortality. This finding implies that neonatal mortality and the proportion of neonatal deaths in the first 24 hours may depend on other factors, such as: (i) the quality of the intrapartum and postnatal care; (ii) the characteristics of the infant, such as birth weight; or (iii) socioeconomic factors, such as maternal literacy.

### Limitations

One limitation of our analysis is that our estimates of neonatal mortality were not nationally representative because the studies were conducted in predominantly rural areas. However, our findings are generalizable to similar, vulnerable populations in low- and lower-middle-income countries because the neonatal mortality rates we observed were not influenced by averaging across different population types. Another limitation is the variation between studies in the surveillance carried out to identify neonatal deaths. Although frequent home visits were made during the neonatal period at most sites, only one or two visits took place in Ghana and India. However, the dates and times of births and deaths were confirmed at all sites by verbal autopsies conducted by experienced field workers within three months of the death. These field workers spent one or two hours with families to determine the circumstances surrounding the death and its timing. Although there is a possibility that the time of death was recalled incorrectly, recall bias is substantially greater in surveys like Demographic and Health Surveys, where the interview may take place several months or even years after a neonatal death. Another limitation of our study is that the exact time of death was not available in Pakistan, where the date of birth was used to determine whether the death occurred within the first 24 hours of life. Finally, in Zambia, pregnant women enrolled at health-care facilities during antenatal visits and during community outreach programmes – they were not identified by routine community-based surveillance.

In conclusion, our findings suggest that maternal and child health programmes in low- and lower-middle-income countries should focus more on preventing deaths within the first 24 hours of life. This will require access to high-quality care during labour and immediately after birth. Several possible life-saving interventions are effective, cost little and are relatively simple to provide in most settings: (i) resuscitation of babies who are not breathing at birth; (ii) antenatal corticosteroid administration to women in premature labour, as described in recent WHO guidelines;^11^ and (iii) early kangaroo care.^1^ It has been estimated that better and more readily available preconception, antenatal, intrapartum and postnatal interventions could reduce neonatal mortality by 71% and reduce the number of stillbirths by 33% by 2025.^12^ Although great strides were made towards achieving a two-thirds reduction in child mortality between 1990 and 2015, with 19 000 fewer children dying each day in 2015 than in 1990,^13^ the comprehensive implementation of high-quality and effective obstetric and early newborn care should still be high on the agenda.
